# The effect of progressive resistance training on lean soft tissue mass in head and neck cancer patients during concomitant chemoradiotherapy: the DAHANCA 31 randomized controlled trial

**DOI:** 10.2340/1651-226X.2026.45545

**Published:** 2026-07-10

**Authors:** Camilla K. Lonkvist, Simon Lønbro, Anders Vinther, Bo Zerahn, Eva Rosenbom, Mads Vansted Svart, Morten Lyng Høgild, Hanne Primdahl, Julie Gehl

**Affiliations:** a Department of Oncology, Herlev and Gentofte Hospital, University of Copenhagen, Herlev, Denmark; b Department of Experimental Clinical Oncology, Aarhus University Hospital, Aarhus, Denmark; c Department of Public Health, Section for Sport Science, Aarhus University, Aarhus, Denmark; d Department of Physiotherapy and Occupational Therapy and Hospital, Herlev and Gentofte Hospital, University of Copenhagen, Herlev, Denmark; e Department of Clinical Medicine, Faculty of Health and Medical Sciences, University of Copenhagen, Copenhagen, Denmark; f Department of Clinical Physiology and Nuclear Medicine, Herlev and Gentofte Hospital, University of Copenhagen, Herlev, Denmark; g Nutritional Research Unit, Herlev and Gentofte Hospital, University of Copenhagen, Herlev, Denmark; h Department of Internal Medicine, Regional Hospital Horsens, Horsens, Denmark; i Steno Diabetes Center Aarhus, Aarhus University Hospital, Aarhus, Denmark; j Department of Clinical Medicine, Aarhus University, Aauhus, Denmark; k Medical Research Laboratory, Department of Clinical Medicine, Aarhus University Hospital, Aarhus, Denmark; l Department of Oncology, Aarhus University Hospital, Aarhus, Denmark; m Department of Clinical Oncology and Palliative Care, Zealand University Hospital, Roskilde, Denmark

**Keywords:** resistance training, head and neck neoplasms, chemoradiotherapy, body composition, muscle strength, physical functional performance

## Abstract

**Background and purpose:**

Head and neck cancer patients undergoing concurrent chemoradiation treatment (CCRT) lose a significant amount of lean soft tissue mass (LSTM) reducing muscle strength and performance. Progressive resistance training (PRT) may mitigate this loss and improve muscle strength and performance. The purpose was to investigate the effects of PRT initiated at CCRT onset on LSTM, muscle strength, and functional performance compared with usual care.

**Patients/material and methods:**

Patients were randomized to 12 weeks of PRT (36 sessions) or usual care with no exercise (control group [CON]). At baseline, after 6 and 12 weeks, 6 and 12 months, Dual-Energy Absorptiometry evaluated total body mass (BM), LSTM, and fat mass (FM); 1 repetition maximum (1RM) chest press and leg press test assessed maximal muscle strength and functional performance (30 seconds chair-rise; 30 seconds arm curls; stair climb) was evaluated. Dietary intake was registered as were Quality of Life questionnaires European Organization for Research and Treatment of Cancer Quality of Life Questionnaire (QLQ) C30 and QLQ H&N-35. A sample size of 72 patients (36 per group) was determined a priori with LSTM as the primary endpoint.

**Results:**

Fifty patients were included (required sample size was not met), 25 in each group. From baseline to 12 weeks, patients in the PRT group lost 8.6 ± 1.0 kg BM (10%), 3.4 ± 0.6 kg LSTM (6%), and 5.2 ± 0.8 kg FM (21%). This was not significantly different from the CON group losing 7.4 ± 1.0 kg BM (9%), 3.4 ± 0.6 kg LSTM (6%), and 4.0 ± 0.8 FM (17%). Likewise, 1RM decreased equally in both groups. Chair rise and arm curl performance improved significantly more in PRT compared with in CON (*p* < 0.05).

**Interpretation:**

In this study PRT initiated at CCRT onset did not attenuate loss of LSTM or muscle strength. However, PRT did result in significantly increased functional performance.

## Graphical abstract

**Figure:**
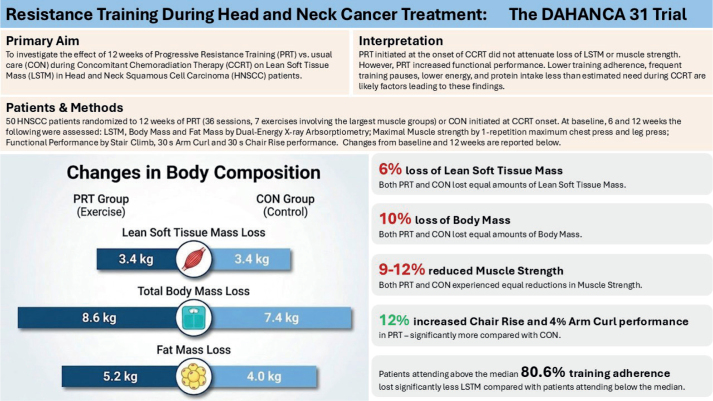

## Introduction

Head and neck squamous cell carcinoma (HNSCC) patients undergoing concurrent chemoradiation treatment (CCRT) encounter severe loss of lean soft tissue mass (LSTM) [1–5]. Recently, we reported a 9.3% (4.7 kg) decline from pre-CCRT until 2 weeks post-treatment [1]. Causes are mainly treatment-induced side-effects such as ulcerations of the digestive tract, pain, and dysphagia [1, 6]. Pre-radiotherapy skeletal muscle depletion is associated with overall and disease specific survival and post-radiotherapy depletion is associated with overall survival and locoregional control too [7]. Furthermore, LSTM loss is significantly associated with reductions in maximal muscle strength and functional performance [1, 8].

The DAHANCA 25 trials demonstrated that 12 weeks of progressive resistance training (PRT) initiated 2 months post-CCRT was feasible for HNSCC patients [9]. It effectively restored LSTM, muscle strength, and functional performance to levels of healthy aged-matched subjects [8, 10]. This therapeutic approach, as opposed to prophylactic intervention, permits LSTM loss and its associated negative consequences. Research on the effect of exercise during treatment on LSTM in HNSCC patients is limited to few heterogenic studies, with varying methodological approaches and findings [11]. In a non-controlled pilot study in 12 HNSCC patients, we demonstrated the feasibility of PRT initiated at the beginning of CCRT and continuing 6 weeks post-treatment [12]. After an initial LSTM reduction occurring during the course of CCRT, LSTM increased after CCRT when PRT was continued. Similar findings were observed for muscle strength and functional performance. However, randomized, controlled investigations are needed to learn whether PRT during CCRT is able to reduce LSTM loss.

The aim of this study was to investigate the effect on LSTM of 12 weeks of PRT initiated at CCRT start in HNSCC patients compared with a control group receiving usual care. Secondly, the effects on maximal muscle strength, functional performance, and quality of life (QoL) were investigated. Exploratively, the more long-term changes after PRT on the same endpoints 6 and 12 months post-treatment were also assessed.

It was hypothesized as well as considered clinically relevant that PRT would attenuate the loss of LSTM during the 12 weeks of training by a minimum of 25% compared with usual care. This was based on pilot study’s findings [12], the DAHANCA 25B post-treatment RCT [10] and protocol considerations described earlier [13].

## Patients/material and methods

The methodological approach including the PRT program is previously described in detail [13]. This study was a randomized, controlled, stratified, and parallel-grouped study. Patients were randomized 1:1 to either a 12-week PRT program (PRT) or control group (CON) following a stratification by site (Herlev/Aarhus), sex (male/female), tumor p16-status (positive/negative), and Body Mass Index (BMI) (below/above 30). The DAHANCA (Danish Head and Neck Cancer Group) secretary (www.dahanca.dk) performed the randomization using a software randomization file.

### Patients

Patients were recruited from the Departments of Oncology at Herlev and Gentofte Hospital, University of Copenhagen and Aarhus University Hospital. Eligible patients were newly diagnosed with primary HNSCC scheduled for curatively intended, concomitant, accelerated chemoradiotherapy. An oncologist provided written and verbal trial information during patients’ initial visit and patients were given time to consider participation before signing written consent. Those who withdrew consent within the first week after inclusion were replaced as per protocol.

Study inclusion criteria were: (1) histologically verified primary HNSCC of the oral cavity, oropharynx, hypopharynx, larynx, or in lymph nodes of the neck from an unknown primary tumor; (2) candidate for curatively intended CCRT; (3) performance status (PS) 0–1 (Eastern Cooperative Oncology Group Performance (ECOG); (4) age ≥ 18 years; (5) signed informed consent. Exclusion criteria were: (1) BMI < 20.5; (2) comorbidity, psychological, social, or geographical conditions potentially interfering with protocol adherence or endpoint assessment (3) tonsillectomy within 1 week before inclusion; (4) insufficient bone marrow function (hemoglobin < 6 mmol/L, leucocytes < 2.5 × 109/L, or thrombocytes < 50 × 109/L; (5) diastolic blood pressure < 45 or > 95, resting heart rate > 100; (6) signs of ischemia on electrocardiogram; (7) pregnancy.

All patients received CCRT based on DAHANCA guidelines, 66–68 Gy, in 2 Gy fractions, 6 fractions/week, with concurrent nimorazole (1200 mg/m^2^) before each fraction (1000 mg/m^2^ for same day second fraction), and concurrent weekly cisplatin (40 mg/m^2^, max. 70 mg). Prophylactic antiemetics, including prednisolone, were administered as part of treatment.

Dietary counseling was provided by a clinical dietician or nurse at the beginning of CCRT and by nurses during treatment to reduce energy deficiency and weight loss. The number of patients needing tube feeding and the duration of the tube feeding were registered.

### Assessment of body composition, maximal muscle strength and functional performance

All methods below have been described in detail previously [13]. Primary and secondary endpoints were evaluated at baseline (T0), following 6 (T1) and 12 weeks (T2) as well as after 6 (T3) and 12 months (T4) post-treatment initiation. Evaluations were standardized between sites and personnel involved in primary endpoint assessment were blinded to group allocation. The primary endpoint LSTM as well as fat mass (FM), bone mineral content (BMC), and body mass (BM) (BM = LSTM + FM + BMC) were measured using Dual energy X-ray Absorptiometry (DXA) – GE Lunar iDXA (GE Health Care Technologies, Chicago, US.) at the Herlev site and Hologic QDR series (Hologic Inc., Massachusetts, US) at the Aarhus site. One repetition maximum (1RM) unilateral leg press and chest press in conventional training equipment evaluated lower and upper body maximal muscle strength. The 30 seconds chair stand test, 30 seconds arm curl test, and maximal stair climbing performance evaluated functional performance. All methods of endpoint assessment are validated and used previously in Danish HNSCC patients [1, 9, 10, 12].

### Estimation of energy expenditure, energy and protein intake

Total energy and protein intake were estimated from 24-hour recall questionnaires filled out once per week throughout CCRT, 2 weeks post-treatment, and at 2, 6, and 12 months follow-up. Estimated total energy and protein need were derived (by CL using Microsoft Office Excel 2019 software) from calculations described previously [13]. To support adequate energy intake on training days and minimize the risk of a catabolic state, patients in the PRT group were provided with a post-exercise protein supplement (Fresubin 2 kcal 200 mL (Mediq) or Nutridrink Compact, 125 mL (Nutricia)).

### Assessment of QoL

The European Organization for Research and Treatment of Cancer (EORTC) QoL questionnaires, QLQ-C30 and QLQ-H&N-35 assessed measures of QoL and have been used frequently in exercise studies in Danish HNSCC patients [8].

### Biological samples

Blood samples and biopsies from m. vastus lateralis were also collected and findings will be reported in a separate manuscript.

### PRT and control

PRT was initiated at the onset of CCRT and comprised 3 weekly sessions over a 12-week period (36 sessions in total), extending approximately 6 weeks beyond CCRT end. Sessions missed due to clinical obligations or public holidays, but not for personal reasons or fatigue, were rescheduled. Patients unable to participate in planned sessions due to treatment side-effects were encouraged to do two home-based exercises (backward lunges and push-ups) daily until returning to supervised training. All sessions in the initial 6 weeks were supervised by a physiotherapist (Herlev site) or an experienced exercise physiology student (Aarhus site) in hospital training facilities. The final 6 weeks were conducted and supervised at the hospital facilities when possible, or at commercial training facilities near the patients’ residence, but with one weekly session conducted at the hospital. Training attendance and – progression were registered in training logs.

The PRT program entailed seven exercises performed in conventional equipment (Technogym, Gambettola, Italy) and included chest press, low row (Herlev) or lateral pull down (Aarhus), hamstring curls, knee extension, leg press, abdominal crunches (Herlev) or sit-ups (Aarhus) and back extensions. Following an introductory first week, intensity and volume progressed from two to three sets at 15–8 repetitions maximum (RM).

No exercise restrictions were made for patients in CON and no organized training was offered. Leisure time physical activity level was estimated by the Physical Activity Scale (PAS) questionnaire [14].

### Statistics

The sample size calculation of 72 patients randomized 1:1 corresponding to 36 in each group is described previously [13]. Linear mixed effects model analyses evaluated possible time and group interactions of endpoints, and post hoc tests (Student’s *t*-tests) evaluated specific time and group differences. In certain instances, with no significant time and group interaction, post hoc tests were performed to investigate within-group changes in order to document clinically relevant endpoint changes during the course of treatment. Data from the EORTC QLQ-C30 questionnaires were assumed to be ordinally distributed. Thus, unpaired Wilcoxon–Mann–Whitney tests analyzed group differences and paired Wilcoxon signed-rank test analyzed changes over time within groups. Intention to treat analyses were applied, including all patients completing endpoint assessment regardless of PRT adherence. Endpoints were tested statistically using a 5% level as significant and 5–10% as a trend. Unless specified otherwise, data are presented as mean values ± SEM. STATA version 19 was used for all analyses (StataCorp SE, Texas, US).

## Results

**Figure 1:**
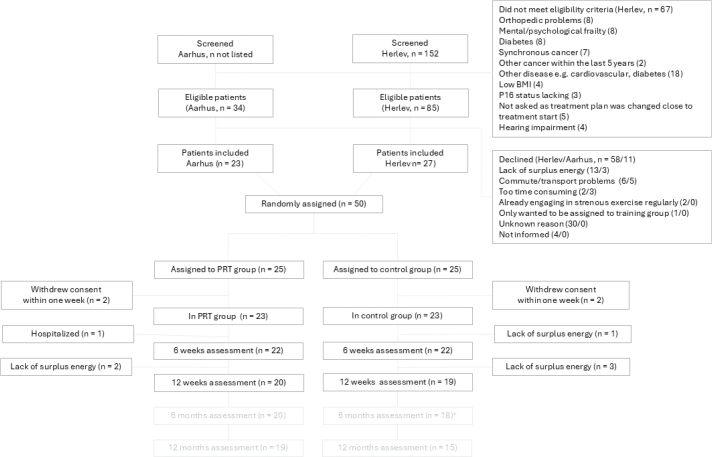
Flow diagram of the study.

From September 25th 2015 to December 2018, 50 of the expected 72 patients were included with last visit of the last patient in January 2020. Due to organisational changes and staff limitations, the Aarhus site ceased inclusion prematurely after 2 years as did the Herlev site in December 2018. Twenty-seven patients were included at Herlev site and 23 patients at Aarhus site (Figure 1). Screening logs were available from Herlev site but not from Aarhus site due to organizational changes. From the Herlev site, 85 patients were eligible and 58 declined to participate. The two centers treat the same number of patients annually, why similar eligibility numbers are estimated between sites. Twenty-five patients were included in each arm of the study and of these 39 remained in the study with, respectively, 20 patients in PRT and 19 in CON. There was no difference in drop-out rate between PRT (n = 5), and CON (n = 6) and the predominant reasons were withdrawal of content within the first week (*n* = 4), and lack of surplus energy to proceed (*n* = 6). Overall, the median age of randomized patients was 55 years; 76% of patients were male; 94% of all patients were diagnosed with tumors of the tonsils or oropharynx and 87% of tumors were p16 positive. See Table 1 for all characteristics of the 23 patients randomized in each group that did not withdraw within the first week (Figure 1).

**Table 1:** Demographic characteristics of the 46 included patients.

	Total	PRT	CON
Patients included	46	23	23
Age, median years (range)	55 (38–79)	58 (49–65)	55 (38–79)
Sex			
Male	35 (76%)	18	17
Female	11 (24%)	5	6
Primary site			
Oral	1 (2%)	1	0
Tonsil	15 (33%)	9	6
Oropharyngeal	28 (61%)	13	15
Nasopharyngeal	1 (2%)	0	1
Hypopharyngeal	1 (2%)	0	1
Tumor p16 status			
Positive	40 (87%)	20	20
Negative	6 (13%)	3	3
Clinical stage			
I	30	18	12
II	4	1	3
III	6	1	5
IVA	4	3	1
IVB	1	0	1
Unknown	1	0	1
Performance status (ECOG scale)			
0	44 (96%)	22	22
1	2 (4%)	1	1
Smoking status			
Never	17	10	7
Former	20	10	10
Current	9	3	6
Level of self-reported physical activity at baseline*			
Low	8	3	5
High	34	17	17
Missing	4	3	1
BMI			
≥ 30	7 (15%)	3	4
25.0–29.9	20 (43%)	11	9
< 25	19 (41%)	9	10
Tube administration			
Nasogastric	24 (52%)	12	12
PEG	6 (13%)	3	3
No tube	14 (30%)	8	6
Missing	2 (4%)	0	2
Days from referral to radiotherapy start			
Median (range)	11 (7–27)	10 (7–27)	11 (7–21)

BMI: Body Mass Index; ECOG: Eastern Cooperative Oncology Group; PRT: Progressive Resistance Training; CON: control.

* Self-reported physical activity below (low) or above (high) 750 MET min/week of moderate and/or vigorous physical activity.

### Body composition

There was no significant group x time interaction for LSTM (*p* = 0.59) but a trend for BM (*p* = 0.053) and FM (*p* = 0.076). Thus, the hypothesized 25% LSTM attenuation in PRT from T0 to T2 was not achieved.

**Figure 2A–C:**
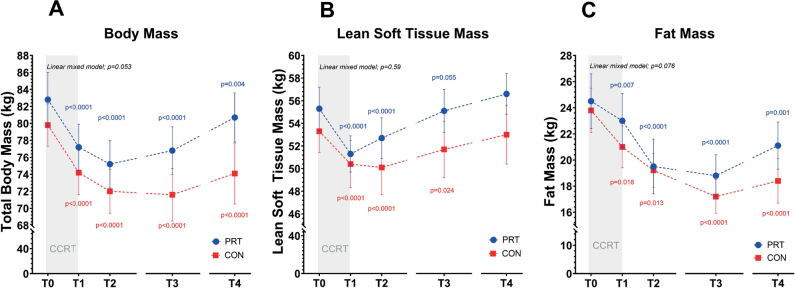
Absolute total body mass (A), lean soft tissue mass (B) and fat mass (C) in kg at T0 (baseline), T1 (post-treatment), T2 (post 12 weeks of PRT or CON), T3 and T4 (6 and 12 months post-treatment). PRT: Progressive Resistance Training; CON: control. *P*-values from linear mixed model analyses are displayed in top left corner whereas blue (PRT) and red (CON) *p*-values indicate statistically significant within-group difference at specific timepoint compared with T0 from post hoc analyses. Grey areas represent the CCRT (concurrent chemoradiation treatment) period. Values presented as mean ± SEM.

As illustrated in Figure 2A–C (and Table 1 in Supplementary Material), analyses estimate of LSTM decreased significantly from T0 to T2 in CON by 3.4 ± 0.6 kg (*p* < 0.0001; 95% CI: 2.2; 4.6 kg) and in PRT by 3.4 ± 0.6 (*p* < 0.0001; 2.2: 4.5 kg). BM decreased significantly from T0 to T2 in CON by 7.4 ± 1.0 kg (*p* < 0.0001; 95% CI: 5.4; 9.4 kg) and in PRT by 8.6 ± 1.0 (*p* < 0.0001; 95% CI: 6.6; 10.6 kg). FM decreased significantly from T0 to T2 in CON by 4.0 ± 0.8 kg (*p* < 0.0001; 95% CI: 2.4; 5.6 kg) and in PRT by 5.2 ± 0.8 (*p* < 0.0001; 95% CI: 3.6; 6.8 kg).

LSTM returned to T0 levels from T3 in PRT and from T4 in CON (Table 1 in Supplementary Material). In both groups, total BM and FM were still significantly lower compared with T0 levels (pre-treatment) at T3 (6 months) and T4 (12 months).

### Maximal muscle strength and functional performance

No significant time x group interactions were observed in 1RM leg press or chest press. From T0 to T2 1RM leg press decreased significantly in CON by 12.0 ± 3.9kg (*p* = 0.02; 95% CI: 1.2; 16.5 kg) and by 15.4 ± 3.9 kg (*p* = 0.003; 95% CI: 3.1; 14.5 kg) in PRT. Similarly, 1RM chest press decreased significantly by 9.2 ± 2.9 kg (*p* = 0.002; 95% CI: 3.4; 15.0 kg) in CON and by 8.8 ± 2.9 kg (*p* = 0.003; 95% CI: 3.1; 14.5 kg) in PRT. According to the linear mixed models, there were group x time interactions for performance in 30 seconds arm curl and chair rise (*p* < 0.05) but not in stair climb performance, and post hoc analyses revealed a significantly larger change from T0 to T2 in chair rise (*p* = 0.005, 95% CI: 0.8; 4.6 repetitions) and 30s arm curl performance (*p* = 0.02, 95% CI: 0.4; 4.5 repetitions) in PRT compared with CON.

### Training adherence and progression

**Figure 3A–B:**
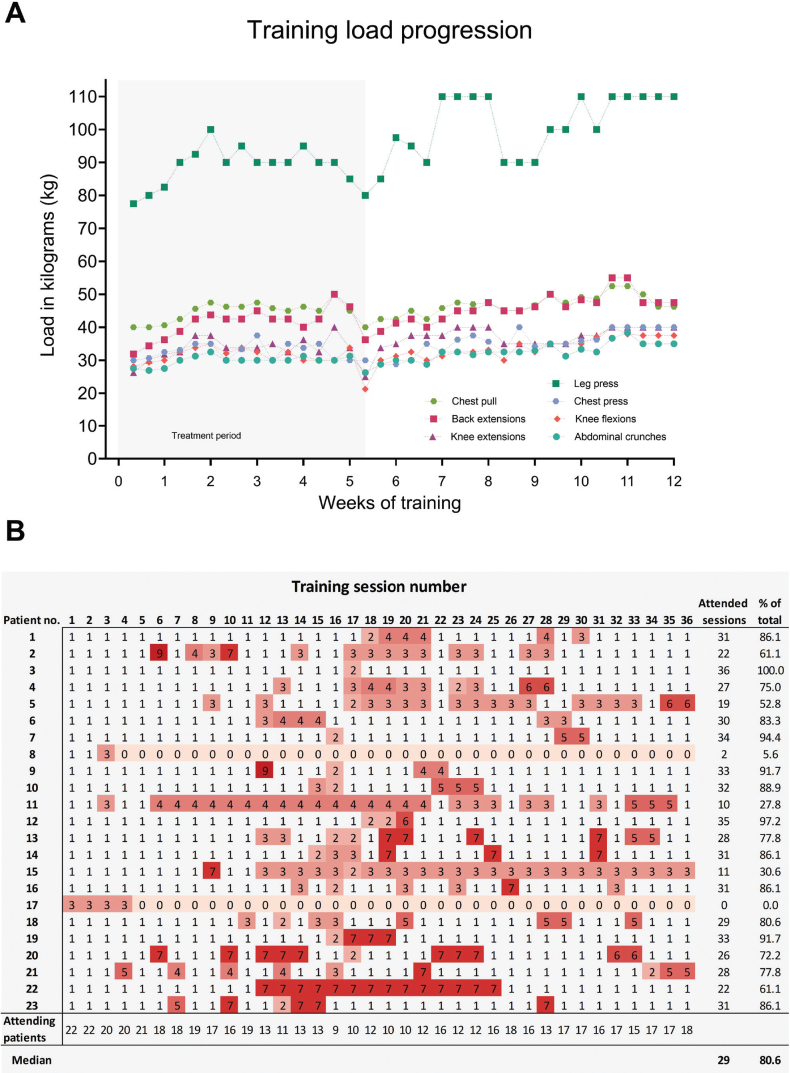
(A) Mean training load progression in kg of the PRT patients in all seven exercises during 12 weeks of training. (B) Training adherence and reasons for missed session of each individual patient in PRT. 0: Patient drop out; 1: Attended; 2: Training replaced by test; 3–9: Not attended due to physical weakness (3), Hospital admission (4), personal reasons (5), other diseases or illnesses (6) and reason unknown (7). A few times it was unclear whether the patient took part in training (9). PRT: Progressive Resistance Training.

Based on training instructor experience, we investigated the significance of PRT attendance on LSTM changes from T0 to T2. As illustrated in Figure 3B, attendance decreased gradually during CCRT, before increasing slowly during the last 4–5 weeks. Patients with a 12 week training attendance lower than the group median of 80.6%, LSTM decreased by 5.1 ± 1.2 kg, a significantly larger loss compared with the 2.2 ± 0.7 kg loss in patients with attendance higher than the median (unpaired Student’s *t*-test; *p* = 0.04; 95% CI: –0.1; –5.7 kg). Figure 3A indicates an increase in absolute training load (in kg) across exercises during the first 2–3 weeks whereafter it leveled off, before increasing slightly the last 4–5 weeks.

The primary registered causes of missed sessions over the entire 12-week training period were weakness (78 sessions), unknown reasons (40 sessions) and hospital admission (33 sessions) (Figure 3B). There were no reports of training-related injuries.

### Nutritional intake

**Figure 4:**
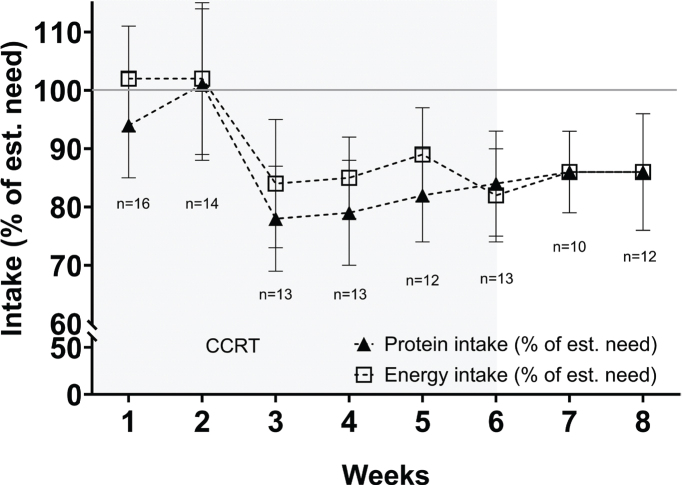
Estimated energy and protein intake from the first week of CCRT to 2 weeks after in the PRT group. Data presented as mean values ± SEM. PRT: Progressive Resistance Training; CCRT: concurrent chemoradiation treatment.

There were no significant group differences in total daily energy intake, protein intake, percent of estimated daily energy and protein need at any time point (*p* > 0.05) (Figure 4 and Supplementary Material Table 2). In PRT, the mean percentage of estimated energy need in the initial 2 weeks of CCRT surpassed 100%, whereas the combined mean of the next 6 weeks decreased by 19% points to 86% (*p* = 0.04; 95% CI: 1.3; 36.8%). In CON, it decreased non-significantly by 8.5% points to 94% (*p* = 0.5; 95% CI: -16; 35.6). Similar non-significant trends were observed in PRT for percentage of estimated protein need decreasing from an average of 98% in the first 2 weeks to 83% as the combined mean for the next 6 weeks.

In the PRT group, explorative analyses (n = 8) revealed a non-significant trend toward greater LSTM loss from T0 to T2 in patients consuming below the median of 92% of their estimated daily energy need (4.5 ± 1.4 kg loss) compared to those above the median (0.4 ± 1.2 kg loss), with a mean difference of 4.0 ± 1.9 kg (p = 0.07; 95% CI: -0.5 to 8.6 kg).The percentage of estimated energy need was calculated as the means of the eight first consecutive weekly reports from T0 to week 8 post-treatment initiation. A non-significant 2.9 ± 2.2 kg larger (*p* = 0.2; 95% CI: -2.4; 8.3 kg) LSTM loss was observed in the patients below the median 88% of estimated protein need (3.9 ± 1.8 kg loss) compared with patients above the median (1.0 ± 1.2 kg loss). The development in estimated energy and protein intake is illustrated in Figure 4.

### Physical activity level and QoL

There were no significant group differences in estimated level of moderate and vigorous leisure time physical activity at any time point. Overall, the PAS answer rate ranged between 39–65% during all 12 weeks (Supplementary Material, Figure 4).

There were no significant time x group interactions in any EORTC QLQ C30 and QLQ H&N-35 variables except Physical Functioning, which declined significantly more in CON from T0 to T1 (*p* < 0.05) and all variables except Emotional Functioning declined from T0 to T1 in both groups (*p* < 0.05) (See Supplementary Material Figure 1–3).

## Discussion and conclusion

This study did not find that 3 weekly sessions of PRT initiated alongside CCRT would ameliorate the loss of LSTM by 25% compared with usual care. Both CON and PRT experienced substantial LSTM losses of 6% during treatment, and despite a non-significant 1.4% LSTM increase in PRT and a numerical non-significant 0.2% decrease in CON from T1 to T2, this group difference was insignificant (Figure 2B). The T0–T2 LSTM loss across groups is in line with our previous one-armed (PRT intervention) pilot study observations [12], but in contrast to Zhao et al. [15], who used a similar design but reported unchanged levels of LSTM. The multimodal training approach comprising lower resistance training load and volume as well as a smaller cohort could explain their findings.

In this study, 3 weekly sessions were planned since this is both feasible and can improve LSTM effectively [10]. The median training attendance in PRT was 80.6%, which is lower compared to previous studies [10, 12] and potentially leaves training volume sub-optimal to attenuate LSTM loss. In support, higher training attendance was associated with a lower LSTM reduction and patients above median attendance experienced a significant lower loss compared with patients with attendance below the median. Furthermore, the total number of missed training sessions among all included patients increased from 51 during the initial 12 sessions to 118 in next 12 sessions and decreased again to 78 sessions in the final 12 sessions. The increase after the initial third correlates with the onset of radiation-induced side-effects 2–3 weeks into radiotherapy [6, 16]. We acknowledge that the association analyses rule out causal conclusions and that treatment complications, PS and etc. may blur the association between training adherence and adaptation.

**Figure 5A–E:**
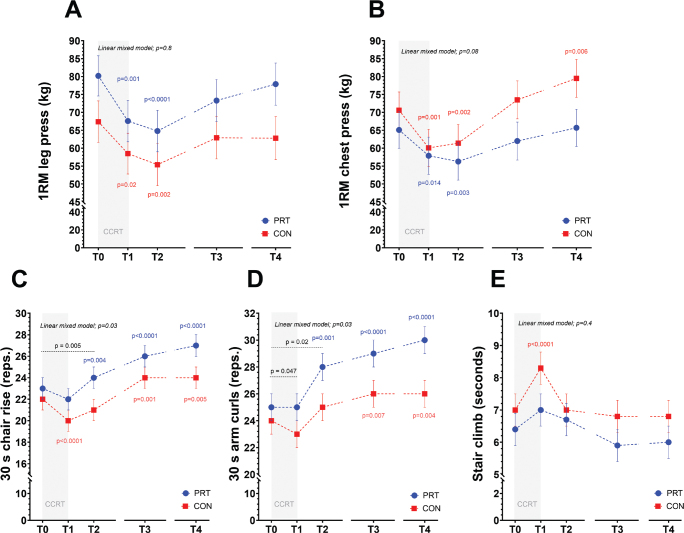
Maximal 1RM (1 repetition maximum) leg press in kg (A), 1RM chest press in kg (B), 30 seconds chair rise (repetitions) (C), 30 seconds arm curls (repetitions) and stair climbing performance (seconds) at T0 (baseline), T1 (post-treatment), T2 (post 12 weeks of PRT or CON), T3 and T4 (6 and 12 months post-treatment). PRT: Progressive Resistance Training; CON: control. *P*-values from linear mixed model analyses are displayed in top left corner whereas blue (PRT) and red (CON) *p*-values indicate statistically significant within-group difference at specific timepoint compared with T0 from post hoc analyses. Grey areas represent the CCRT (concurrent chemoradiation treatment) period. Values presented as mean ± SEM.

Lower training volume and longer breaks in adherence likely compromise training load progression (Figure 3A), underlining that overall load progression leveled off in periods with low adherence. Consequently, 1RM leg press and chest press decreased significantly in PRT from T0 to T1 (Figure 5A and B) in the period with lowest adherence, whereafter it stagnated from T1 to T2 during a period where adherence increased.

Patient energy and protein intake decreased non-significantly to levels below estimated need after the initial few weeks (Figure 4), suggesting that treatment side-effects also anchor a catabolic energy state hindering protein synthesis and consequently muscle mass maintenance [17].

Collectively, these observations are new and of great value, since they argue that a higher patient training adherence rate and before volume so that the sentence is volume as well as adequate dietary intake could ameliorate the loss of LSTM. However, how much larger the training volume needs to be, remains unresolved. Moreover, the fact that training adherence drops drastically at the onset of treatment side-effects and remains lower for several weeks due to weakness and hospital admittance points to the problem that HNSCC patients in general are challenged in upholding a necessary training volume during CCRT. In the DAHANCA 25B RCT [10], we found a significant LSTM increase following PRT (95% adherence) initiated 2 months post-treatment end where the most severe side-effects had disappeared [10]. Consequently, in HNSCC patients PRT timing is likely very important.

There was a time x group interaction in some estimates of functional performance – 30 seconds chair rise and arm curls – that were not impaired in PRT from T0 to T1, whereas 30 seconds chair rise decreased in CON. Furthermore, both measures increased from T1 to T2 in PRT but not in CON. These findings cannot be explained by changes in LSTM or muscle mass alone, even though correlations between these have been reported in HNSCC patients previously [8]. Likely explanations are that improvements in motor- and neuromuscular function that occur with increase in everyday activities may have a significant impact on these variables. Self-reported questionnaires have inherent limitations in detecting precise variations in physical activity; nevertheless, our analysis of these data demonstrated no group differences.

Similar to previous findings [18, 19], total BM remained lower in both groups during the first 12 weeks but also beyond 6 and even at 12 months compared with pre-CCRT levels [2, 3]. After the first 6 weeks, this was primarily driven by a continuous loss of FM even as late as 6 months post-CCRT (See Supplementary Material Table 1) [20].

We observed no time x group interactions in any QoL or symptom data (except Physical Functioning) from the questionnaires, which was expected since the sample size was not calculated based on changes in these endpoints and since several life events other than participating in training intervention likely affect these outcomes.

### Strengths and limitations

This study is to our knowledge the largest RCT investigating the effect of PRT during treatment in HNSCC patients. The robust methodological approach of the study ensured reliable evaluations of the primary and secondary endpoints, comprised a well-planned and evidence-based training intervention and provided data on training adherence and progression. While energy and protein intake estimates helped identify potential dietary confounders, missing data as well as the 24-hour-recall approach likely limits the overall interpretation. The latter approach was chosen to limit the overall patient burden. Questionnaires on physical activity enabled analyses of confounding group differences, however low response rates limit interpretation.

The inclusion of patients was limited to 50 of the planned 72, compromising statistical power and increasing type II error risk. Thus, despite the obvious consequences of lower training adherence, and -progression, and the potential catabolic state of the patients, it cannot be excluded that the numerical trend of LSTM increase from T1 to T2 would come out statistically significant with inclusion of all planned patients. However, reaching the hypothesized goal of 25% reduced LSTM loss seems unlikely even with enough statistical power and the similar significant declines in LSTM from T0 to T1 observed in both groups strongly indicates that PRT without a positive energy balance during this period does not ameliorate the initial loss of LSTM.

As in a vast majority of exercise oncology studies, the study is subject to selection bias since patients had high performance level, were mostly p16 positive and presented low-stage disease. Thus, the findings cannot be extrapolated directly to HNSCC patients with poorer performance level and prognosis as observed in advanced stage cancer and p16 negative patients [21].

Twelve weeks of PRT initiated at CCRT start did not mitigate loss of LSTM and muscle strength in HNSCC patients compared with usual care. Lower training adherence, frequent training pauses, lower energy, and protein intake than estimated need during CCRT are likely factors leading to these findings. Thus, robust information is provided on the difficulties of HNSCC patients to adhere consistently to training and to maintain a positive energy balance during and immediately after CCRT. Initiating PRT at a later stage, when these factors no longer negatively affect the response to training may be more beneficial [10]. Furthermore, more effective dietary interventions to ameliorate the negative energy balance should be investigated. These points should be considered thoroughly when planning PRT interventions for HNSCC patients. Notably, chair rise and arm curl performance were improved after PRT.

## Supplementary Material



## Data Availability

The data of this study are not publicly available due to the General Data Protection Regulation (GDPR) to ensure privacy and security of personal data.
