# Supplementary material for: The effect of progressive resistance training on lean soft tissue mass in head and neck cancer patients during concomitant chemoradiotherapy: the DAHANCA 31 randomized controlled trial
Source: Acta Oncol. 2026 Jul 10;65:45545. doi: 10.2340/1651-226X.2026.45545 (PMC13358791; doi:10.2340/1651-226X.2026.45545)
Supplement: Supplementary file 1 [file AO-65-45545-s1.pdf]

Supplementary material has been published as submitted. It has not been copyedited, or typeset by Acta Oncologica

Supplementary Material

Fig.1. QoL EORTC QLQ C30

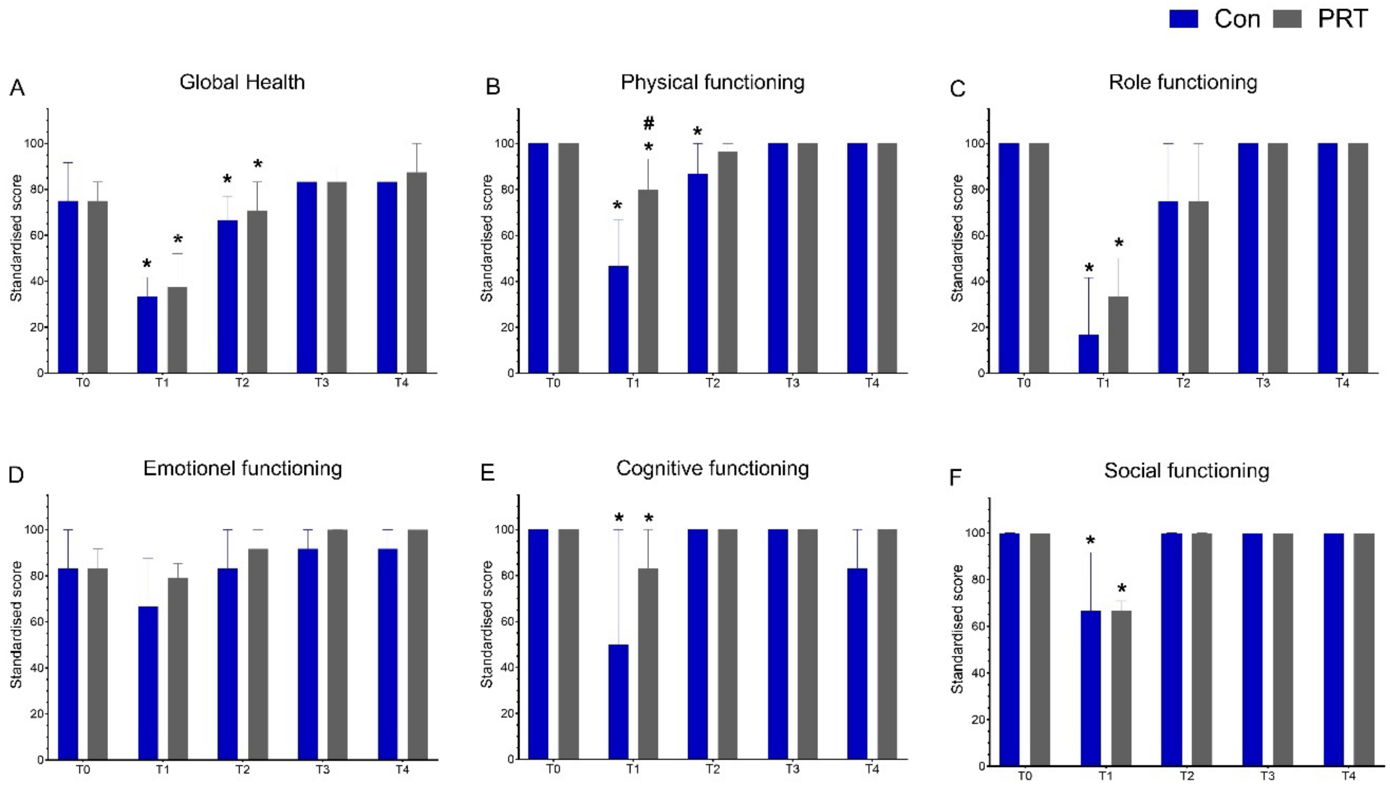

Fig 1. Quality of Life (QoL) scores from the EORTC QLQ C30 questionnaire at baseline (T0), post-treatment (6 wks, T1)), post intervention (12 wks, T2) (PRT, grey bars) or control (CON, blue bars) period, 6 (T3) and 12 months post-treatment start (T4). Higher scores represent higher levels of QoL and function. * Indicates statistically

significant difference ($p<0.05$) compared with T0 within group. # Indicates statistically significant group difference in change from T0 ($p<0.05$). Data presented as median standardized values with interquartile range.

Fig. 2. QoL EORTC QLQ C30

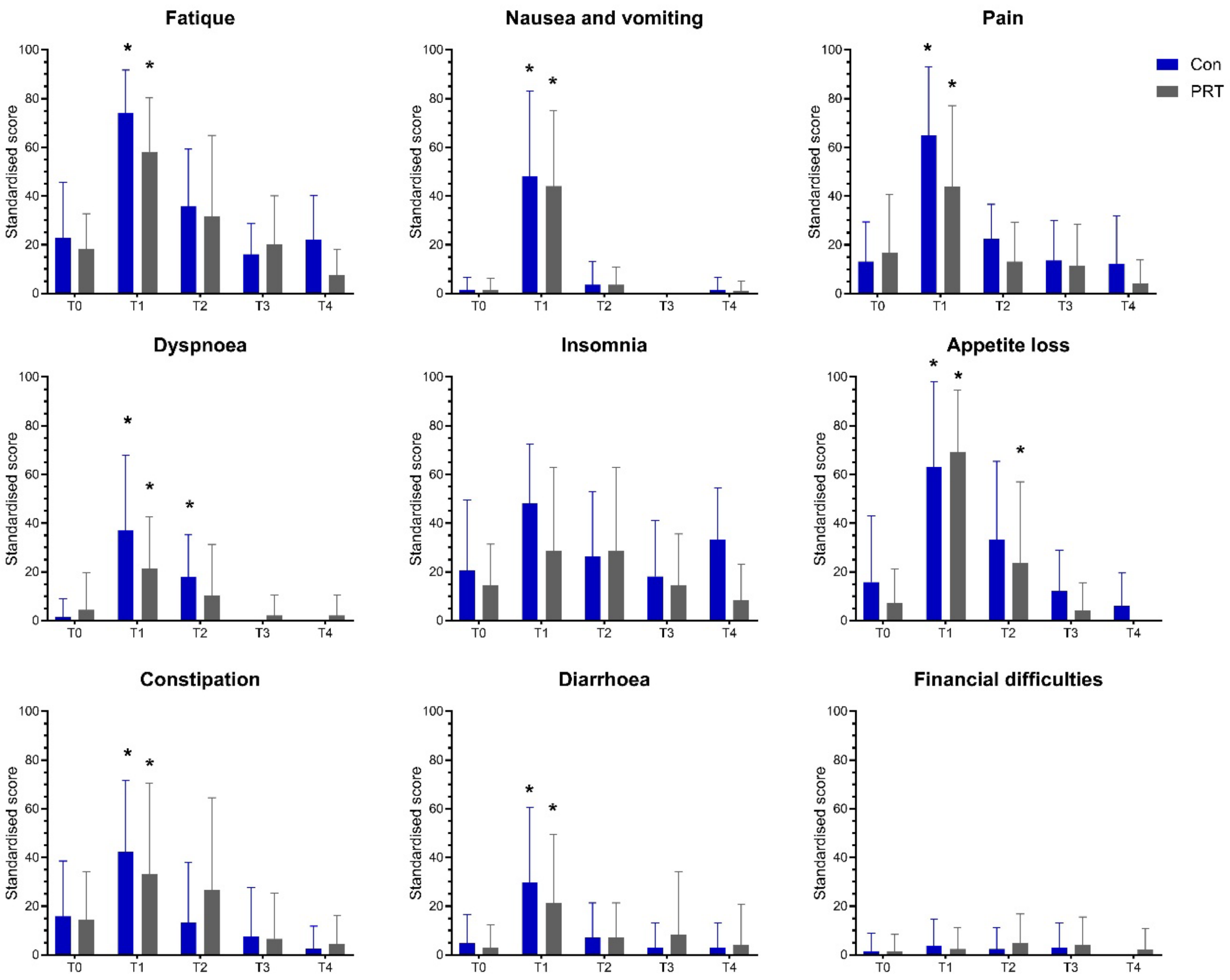

Fig. 2. Quality of Life (QoL) symptom scores from the EORTC QLQ C30 questionnaire at baseline (T0), post-treatment (6 wks, T1)), post intervention (12 wks, T2) (PRT, grey bars) or control (CON, blue bars) period, 6 (T3) and 12 months post-treatment start (T4). Higher scores represent higher levels of QoL and function. * Indicates statistically significant difference (p<0.05) compared with T0 within group. Data presented as mean values with SD.

Fig. 3 - QoL EORTC QLQ H&N-35

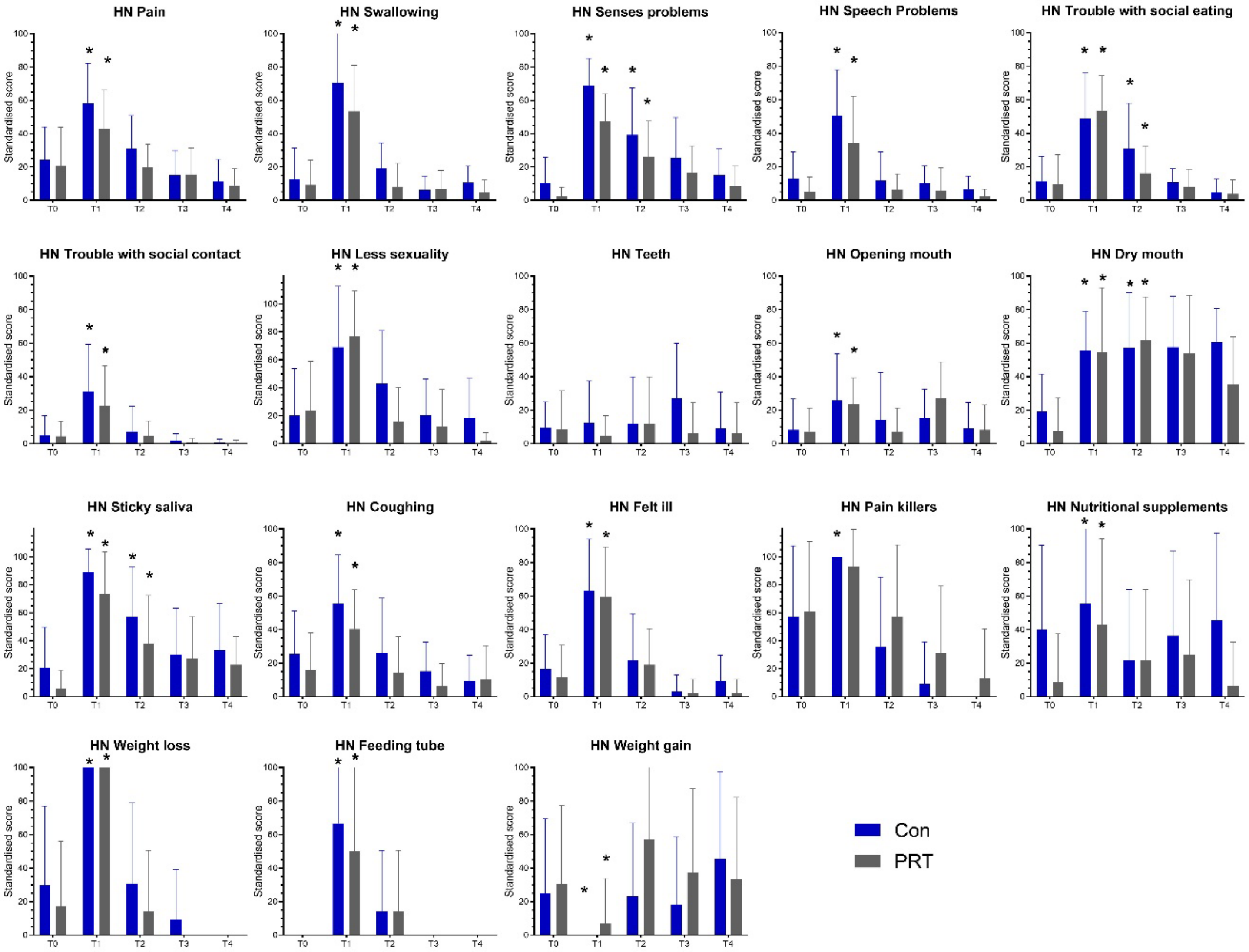

Fig. 3. Quality of Life (QoL) symptom scores from the EORTC QLQ-H&N-35 questionnaire at baseline (T0), post-treatment (6 wks, T1)), post intervention (12 wks, T2) (PRT, grey bars) or control (CON, blue bars) period, 6 (T3) and 12 months post-treatment start (T4). Higher scores represent higher levels of QoL and function. * Indicates statistically significant difference (p<0.05) compared with T0 within group. Data presented as mean values with SD.

Fig.4. Self-reported Moderate and vigorous physical activity.

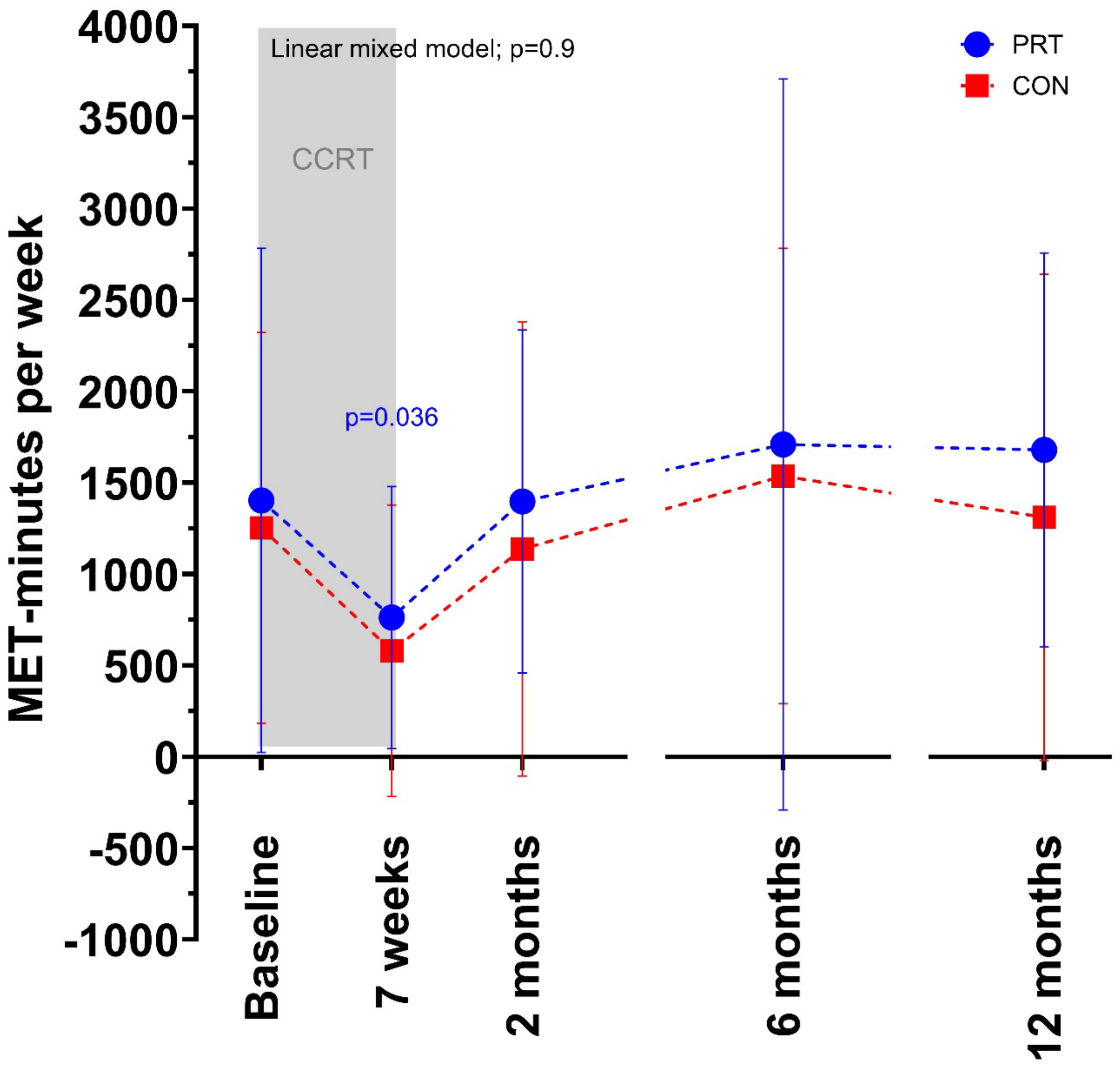

Fig. 4.: Self-reported moderate and vigorous physical activity estimated in MET (Metabolic Equivalent of Task) minutes per week. Physical Activity Scale (PAS) questionnaire pre-treatment (baseline), post-treatment (7 weeks), 2, 6 and 12 months post-treatment. PRT: Progressive Resistance Training; CON: Control. Data presented as mean with SEM. P-value indicate significant difference from baseline.
